# Tailored Implementation of Evidence-Based Practice for Patients with Chronic Diseases

**DOI:** 10.1371/journal.pone.0101981

**Published:** 2014-07-08

**Authors:** Michel Wensing, Elke Huntink, Jan van Lieshout, Maciek Godycki-Cwirko, Anna Kowalczyk, Cornelia Jäger, Jost Steinhäuser, Eivind Aakhus, Signe Flottorp, Martin Eccles, Richard Baker

**Affiliations:** 1 Radboud University Medical Centre, Nijmegen, The Netherlands; 2 Medical University of Lodz, Lodz, Poland; 3 Heidelberg University Hospital, Heidelberg, Germany; 4 Norwegian Knowledge Centre for the Health Services, Oslo, Norway; 5 Newcastle University, Newcastle upon Tyne, United Kingdom; 6 University of Leicester, Leicester, United Kingdom; Canadian Agency for Drugs and Technologies in Health, Canada

## Abstract

**Background:**

When designing interventions and policies to implement evidence based healthcare, tailoring strategies to the targeted individuals and organizations has been recommended. We aimed to gather insights into the ideas of a variety of people for implementing evidence-based practice for patients with chronic diseases, which were generated in five European countries.

**Methods:**

A qualitative study in five countries (Germany, Netherlands, Norway, Poland, United Kingdom) was done, involving overall 115 individuals. A purposeful sample of four categories of stakeholders (healthcare professionals, quality improvement officers, healthcare purchasers and authorities, and health researchers) was involved in group interviews in each of the countries to generate items for improving healthcare in different chronic conditions per country: chronic obstructive pulmonary disease, cardiovascular disease, depression in elderly people, multi-morbidity, obesity. A disease-specific standardized list of determinants of practice in these conditions provided the starting point for these groups. The content of the suggested items was categorized in a pre-defined framework of 7 domains and specific themes in the items were identified within each domain.

**Results:**

The 115 individuals involved in the study generated 812 items, of which 586 addressed determinants of practice. These largely mapped onto three domains: individual health professional factors, patient factors, and professional interactions. Few items addressed guideline factors, incentives and resources, capacity of organizational change, or social, political and legal factors. The relative numbers of items in the different domains were largely similar across stakeholder categories within each of the countries. The analysis identified 29 specific themes in the suggested items across countries.

**Conclusion:**

The type of suggestions for improving healthcare practice was largely similar across different stakeholder groups, mainly addressing healthcare professionals, patient factors and professional interactions. As this study is one of the first of its kind, it is important that more research is done on tailored implementation strategies.

## Introduction

The prevalence of chronic diseases is high and rising worldwide [1]. Although evidence-based recommendations for the diagnosis and treatment are available, many patients with these conditions do not receive evidence-based healthcare [2]–[4]. A range of interventions and policies for implementing evidence-based practice have been developed and tested, showing mixed, unpredictable, and overall moderate impacts [5]. Experts have emphasized that strategies for implementing recommended practices need to be tailored to the determinants of practice faced by the targeted individuals and organizations [6]. For instance, a lack of knowledge (a determinant of practice) may be addressed by providing education and lack of priority for a recommended practice (also determinant of practice) by organizating support from organizational or opinion leaders. Tailoring can be done in different ways, varying from a simple group interview with directly involved clinicians to a systematic stepwise approach, which involves a series of studies involving relevant populations. Generating suggestions for strategies that address barriers to change is an important step in tailoring methods, but research evidence on the validity and efficiency of different approaches to tailoring strategies for improving healthcare is scarce [6].

A systematic review of studies evaluating the effectiveness of tailored strategies suggested that these overall had positive, albeit moderate, effects [7]. This review also reported considerable heterogeneity of tailoring methods, which suggested that the validity of different approaches to tailoring is not well established. It is particularly unclear how strategies for improving practice are best generated. A qualitative analysis of evaluations of tailored improvement programs found that the reported determinants of practice and the chosen interventions to address those did not necessarily match up well with each other [8]. For instance, organizational factors requiring change frequently remained unaddressed by the chosen interventions. Some authors have argued for a more systematic approach for planning and managing tailoring strategies, using either a behavior change theory [9] or a pragmatic framework [10]. These authors believe that a systematic and planned approach helps to consider aspects that may otherwise be ignored.

Other authors argued that processes of change in healthcare delivery are complex and socially constructed, so that strategies need to build on the interactions of relevant stakeholders in order to make sense to them [11]. Some have conceptualized implementation of recommended practices as a social process of “normalization”, which can be influenced by strategies such as regulations and sanctions [12]. This perspective suggests that generating tailored strategies for improving healthcare should engage relevant stakeholders in the design and delivery of strategies.

The “Tailored Implementation for Chronic Diseases” project aimed to assess methods for constructing tailored strategies to implement evidence-based practice in healthcare for patients with chronic diseases [13]. For generating strategies to improve practice, it engaged stakeholders in group interviews and, simultaneously, used a pre-defined framework of determinants of practice to guide the group interviews, their analysis and the subsequent choice of interventions for further evaluation [14]. In this paper we report on a thematic content analysis of the items generated by the interviewed stakeholders in five countries. Our primary objective was to explore how the items mapped onto the pre-defined framework of determinants of practice, which guided the group interviews to generate these. In addition, we were interested to compare the items of different stakeholder groups regarding the domains they addressed.

## Methods

### Study design

A pragmatic interview study using brainstorming in groups to generate items was conducted in five countries: Germany, the Netherlands, Norway, Poland, and the United Kingdom. The study (including participant consent procedure) was assessed and approved by ethical committees in each of the five participating countries: Ethics Committee Heidelberg (Germany), Bioethics Committee of the University of Lodz (Poland), Committee for Research in Humans Radboudumc (Netherlands), Regional Committee for Medical and Health Research (Norway), NRES Committee London - Camden & Islington (UK). Participants were invited several days before the meeting (by letter or telephone). Showing up and giving verbal agreement (after full disclosure on the study) at the location and date of the planned interview was taken as informed consent, with some exceptions. In Germany and the UK, participants also gave written informed consent. In the Netherlands, patients gave written informed consent (these data are not used in this manuscript). Data collection took place between September and December 2012. The research was planned in a written protocol, which is available on request from the authors. We followed COREQ criteria as much as possible in reporting on the study [15].

### Setting and research team

The study was part of the international research project, “Tailored Implementation for Chronic Diseases” [13]. The international team of researchers had a background in academic primary care, clinical epidemiology and health services research. Researchers in each country focus on a different clinical condition, but all are linked by being chronic, long term conditions. The clinical foci included chronic obstructive pulmonary disease (Poland), cardiovascular disease (The Netherlands), depression in the elderly (Norway), multi-morbidity (Germany), and obesity (United Kingdom). In these countries, healthcare for these conditions is mostly provided in primary care settings. In each country, the same series of studies was performed, focusing on a chosen set of recommendations for high-quality healthcare in the targeted condition. In the first study, determinants of practice in the care of the targeted condition were identified using a mix of methods to interview stakeholders. In the second study, which provided the data for this paper, stakeholders were invited to provide items for improving these previously identified determinants. The third study comprised five distinct cluster randomized trials of tailored implementation programs, which were designed to address the key determinants of practice that were identified.

### Study population

In each country, a convenience sample of participants was used, which was purposeful with respect to the inclusion of different stakeholder groups. Four groups of four to eight individuals each were convened (any individual was in one group only), using mix of methods to approach potential participants. These methods included random sampling in a defined geographic area, an existing professional network, and targeted invitations to specific individuals. The first contact with a potential participant was often in written format, but occasionally by telephone or face-to-face.

Group 1 comprised health researchers, including members of the project teams and other academics with relevant expertise. Group 2 comprised quality improvement officers, not involved in the project teams, who develop or coordinate continuing education and quality improvement for the targeted patients, professionals or healthcare sector. Group 3 comprised healthcare professionals relevant for the implementation, mainly primary care physicians and nurses. Group 4 comprised representatives from external stakeholder organizations, such as authorities, health insurers, and patient organizations. The targeted individuals were unrelated to the researchers, except for group 1. Groups were planned to be homogenous. In some countries, given their differing roles in caring for patients with chronic diseases, physicians and nurses were interviewed in separate groups. In two countries, patients and relatives were also interviewed, but these data have not been included in this paper. The number of sessions was planned to reach data saturation across stakeholder groups, although not necessarily within each of these groups.

### Group interviews

Whilst the clinical focus of the group interviews differed across the countries, all interviews followed the same procedure. Detailed instruction was provided in the international study protocol. The purpose of the interviews was presented as scientific and relevant for improvement of healthcare. Interviews were organized in a variety of locations, including multipurpose meeting rooms, healthcare centres and universities. The interviews were led by group moderators, who had an (mostly clinical) academic background, were experienced in leading group interviews, and (if necessary) familiarized with the TICD project. They invited participants to contribute their ideas to the design of an intervention to improve healthcare. Each interview started with a general introduction that presented the chosen targets for improvement (3 to 8 specific goals), which had been chosen by the national teams on the basis of analysis of prevailing guidelines and evidence for performance gaps. Data on current performance were presented in the groups to indicate gaps with recommended practice. This was followed by a presentation of a consolidated list of determinants of practice (the same list in each group in a specific country), which was based on a range of empirical studies in earlier phases of the TICD study [14]. Table 1 gives an overview of the determinants of practice, as mapped out onto the pre-defined TICD framework of determinants of practice [14].

**Table 1:** Determinants given to groups mapped out onto the TICD framework domains.

	Multimorbidity (Germany)	Cardiovascular (Netherlands)	Depression (Norway)	COPD (Poland)	Obesity (United Kingdom)
Guideline factors	2	-	2	4	3
Individual health professional factors	13	7	10	9	6
Patient factors	6	2	4	1	3
Professional interactions	1	1	1	2	-
Incentives and resources	10	1	3	8	2
Capacity for organizational change	-	-	3	-	-
Social, political and legal factors	1	-	-	-	-
Total number of determinants of practice	33	11	23	24	14

**Legend**. Figures indicate number of determinants in each domain, which were given at the start of the group interviews in a country.

Using the method of brainstorming, the participants were then invited to provide items for addressing the given determinants to meet the given targets for improvement. The group moderators were instructed to avoid discussions of study designs, research methods or outcome measures. There was no limit to the number of items for improving healthcare, but the discussions were time limited. Spontaneous categorization or prioritization by participants was accepted, but was not actively encouraged by the moderator. The moderator was instructed to check and ask about major omissions regarding goals/determinants and, when present, prompted participants to consider these. The brainstorms were part of a larger group interview, which lasted 105 to 130 minutes in total (median figures per country), except in Poland where they were substantially shorter (median of 67 minutes). A researcher was present to make field notes and provide practical support. The items provided in the brainstorm sessions provided the starting point for a structured interview, which followed directly after the brainstorm (except in Norway, where only brainstorm sessions were done). In this way, the participants had the opportunity to review the items that are used for analysis in this study within the group sessions.

### Data- analysis

The interviews were audio-taped and transcribed by the national study teams (except in Norway, where notes were made during the sessions). Each of the five national study teams prepared transcripts in English for analysis, focused on listing the suggested items. These were transferred into pre-formatted data-files, which listed the items by group. These data-files were prepared by one research team (MW, EH) and validated by the national research teams (Data S1). For each item, we coded independently which of the TICD framework domains [14] was addressed. Items which did not seem to address a particular determinant of practice were excluded from other analysis. Then we categorized the items by domains in the framework and grouped items into themes within each domain. Both the coding and the thematic analysis were done by two researchers (MW, EH), who discussed discrepancies of interpretations and reached agreement on codes and themes. We used Excel to organize the codings and SPSS to provide descriptive figures.

## Results

A total of 115 individuals participated in 22 group interviews and three individual interviews (Table 2). There were no explicit refusals to participate, but response rates in samples were low and some individuals could not participate in the planned meetings for practical reasons. In three countries (Netherlands, Norway, and United Kingdom) two groups of health professionals were formed. In Poland it was not possible to arrange a group meeting with quality improvement officers, so this was replaced by individual interviews with three people. These data were merged as one group. The participants provided a total of 812 items of which 586 addressed a particular determinants of practice (Table 3). The absolute numbers of items differed across stakeholder groups; health professionals provided the highest numbers. The items that did not address a particular determinant (28% of all) were often expressions of high-quality healthcare rather than interventions or policies to implement this. For instance, it was suggested that “healthcare providers should counsel patients” and that “they should follow guidelines”.

**Table 2:** Number of participants in the group interviews (n = 115 individuals).

	Health researchers	Quality improvement officers	Healthcare professionals	Purchasers, authorities, patient organizations	Totals
Multimorbidity in Germany	5	7	4	4	20
Cardiovascular risk management in the Netherlands	7	3	14 **	5	29
Depression in the elderly in Norway	4	5	11 **	6	26
Chronic obstructive pulmonary disease in Poland	4	3 *	4	4	15
Obesity care in the United Kingdom	6	4	9 **	6	25

**Legend.** *individual interviews, ** more than one group interview.

**Table 3:** Domains in the TICD framework addressed by items (n = 812 items).

Groups → Domain addressed:	Health researchers	Quality improvement officers	Healthcare professionals	Purchasers, authorities, patient organizations	Total
Guideline factors	8 (6%)	2 (1%)	6 (3%)	3 (2%)	19 (3%)
Individual professional factors	64 (52%)	74 (54%)	97 (50%)	67 (51%)	302 (52%)
Patient factors	37 (30%)	29 (21%)	64 (33%)	37 (28%)	167 (29%)
Professional interactions	10 (8%)	25 (18%)	19 (10%)	19 (15%)	73 (12%)
Incentives and resources	5 (4%)	6 (4%)	7 (4%)	2 (1%)	20 (3%)
Capacity for organizational change	0 (0%)	1 (<1%)	0 (0%)	3 (2%)	4 (<1%)
Social, political, and legal factors	0 (0%)	1 (<1%)	0 (0%)	0 (0%)	1 (0%)
					
Subtotal of items that target a domain	124	138	193	131	586
Items that did not target a domain (excluded from thematic analysis)	36	57	73	60	226
Total number of items	160	195	266	191	812

**Legend.** Figures refer to number of items by stakeholder group across countries (column percentages between brackets). Percentages refer to subtotal of items that targeted a domain.

The largest number of items addressed individual health professional factors: 52% of all items (Table 3). A high number of items addressed patient factors (29%). Professional interactions were targeted by 12% of the items. Other domains in the TICD framework were addressed by much lower numbers of items for interventions or policies to improve healthcare for patients with chronic diseases. Little variation in the relative proportion of items in specific domains was seen across stakeholder groups, except that quality improvement officers seemed to provide fewer items regarding patient factors.

Table 4 lists the themes, which we identified in the qualitative analysis of the items for improving chronic illness care. The countries from which citations were derived have been coded as follows: GE = Germany; NL = Netherlands; NO = Norway, PL = Poland; UK = United Kingdom. The themes are elaborated in the remaining of this results section.

**Table 4:** Summary of themes in the items for improving healthcare, mapped onto TICD framework domains.

	Themes
Guideline factors	-summary version of guidelines
	-protocols tailored to local conditions
	-more specific clinical recommendations
	-cost analysis included in guidelines
Individual health professional factors	-content of education
	-delivery format of education
	-interventions to enhance the impact of education
	-enhanced use of information technology
	-free up time for healthcare professionals
	-revision of professional roles
	-making organizational changes
	-enhanced collaboration with other care providers
Patient factors	-delivery formats of patient education
	-use of counseling techniques
	-more active patient involvement
	-involvement of relatives and organizations
	-improved accessibility of services
Professional interactions	-local availability of care providers
	-enhanced communication and teamwork
	-involving others in detection of disease
	-use coordination mechanisms
	-change role perceptions regarding collaboration
Incentives and resources	-overall increase of reimbursement for care providers
	-supply of specific staff or devices
	-reimburse specific items
	-financial incentives for patients
Capacity of organizational change	-anchoring in administrative organization
	-more resources
Social, political, and legal factors	-publicity for healthcare providers

### Guideline factors

Examples of determinants of practice in this domain, which were presented in the group interviews, were the availability of clear guidance (UK) and the access to recommendations (PO). Several themes could be identified in the tailored items relating to guidelines for healthcare delivery. A first theme was that guidelines should be made available in a summarized format, for instance “leaflets aimed at clinicians providing clear guidance” (UK). It was also suggested to make summary versions for patients and their relatives. A second theme was that guidelines needed to be translated into tailored protocols, involving local stakeholders. “When a protocol is not available, the practice nurse should be involved in developing a protocol” (NL). A third theme was that guidelines need to be more specific regarding clinical procedures in patients, including referral to other care providers. “Specific guidelines e.g. if BMI>X do Y” (UK). A final theme in this category was that cost analysis needs to be included in the guidelines.

### Individual health professional factors

Presented determinants in this domain included, for instance, awareness of specific services (NO), clinical inertia (NL), lack of routine (GE), trained staff (PO). Tailored items regarding knowledge and skills concerned the (continued) education of physicians and nurses. A first theme concerned the proposed content, which covered communication skills (e.g. motivational interviewing, cognitive behavior therapy), clinical skills (e.g. measuring blood pressure), pharmacological knowledge, use of computerized patient records, and information on options for referring patients (e.g. to a vascular outpatient clinic). A second theme was the format of the education. Items included quality circles, online education, audit and feedback, training with peers, brochures, and role play. A third theme concerned activities or policies to strengthen the impact of the education of healthcare providers. These included financial incentives to take education, a mandate by the chief medical officer, provision of necessary medical devices (e.g. inhalers, PL), coordination with training of other care providers, and organizing the education strategically (“one knowledgeable person per cluster who can advise on guidelines and local services”, UK).

A wide range of tailored items were directly targeted at changing professional behaviors. Many of these related to making organizational changes, which we have conceptualized as strategies that target individual health professional factors. A first theme was enhancing the use of information technology for a range of purposes, including patient records, individual healthcare plans (“electronic accessibility of a care plan for patient and healthcare professionals”, NL), prompts for specific actions, and databases (“list of volunteers who are interested and have knowledge about depression”, NO). A second theme comprised making organizational changes to improve time available for health professionals, including lower number of patients listed in a practice (NO), separate or longer consultations for the targeted condition (UK, NO), and evening interviews (UK, NL). A third theme comprised revision of professionals roles, such as the proposal that only primary care physicians prescribe long-term medication (GE), several proposals to involve pharmacists in drug treatment (GE), enhancing the role of nurses (e.g. “inserting MRC dyspnoea scale to the cards patient's labeled with COPD. To give the scale while waiting for the doctor or check-in on computers.”, PL). A fourth theme comprised a range of organizational changes, including the standardization of clinical instruments (e.g. MRC dyspnoea scale in PL, weight procedures in UK), joint patient record systems (NL), broaden range of services in general practice (NO), organize a separate room for specific clinical procedures (e.g. weighing, UK), and improved continuity of care (“Consistency with the person you are seeing so they can get to know you and your circumstances”, UK). A fifth theme comprised proposals regarding improving collaboration with other care providers and volunteers (NO), including guarantee that a service is available (UK), that sufficient numbers of specialist care providers are present (NO), a lowered threshold for referral (NO, NL), and ideas for coordination of care (“A coordinator in the community who can connect, one office - one website”, NO), system of pathways for patients”, NO). A final theme, mentioned once, was that healthcare professionals should be role models as individuals (e.g. “lose weight”, UK)

### Patient factors

Determinants of practice, which were presented in the groups, included patients' adoption of life style advice (NL), handling of patient records (GE), and cognitive problems (GE). Items for improving chronic illness care, which were targeted at patients, addressed the following themes. A first theme comprised a wide range of ideas on how to provide information to patients, including the use of pictures, repetition, information campaigns, helpdesk, leaflets, different language versions, taped spoken information, group interviews, local television station, text messages, map of local life style programs, and courses. A second theme comprised items for the use of specific counseling techniques, such as goal setting, choosing realistic goals, make a verbal contract with the patient, focus on behavioral consequences (e.g. feeling healthier) rather than health consequences, transparency on “entitled care” (NL), make an individual care plan, and use serious gaming (computer games with educational purposes). A third theme concerned ideas to involve patients more actively: set goals with patients, allow patients to view their own records (e.g. online), encourage patient self-monitoring of risk factors. Specific examples included the items “to give choice who weighs the patient” (UK) and “allowing patients to decide how often they will revisit the clinic will improve attendance rates” (NL). A fourth theme concerned items for involving others, including patients' relatives, peers as buddies, community organizations, work places, and “commercial slimming clubs” (UK). Other items targeting patients concerned reminders and rewards for patients, e.g. financial incentive for using only one pharmacy (GE), active follow-up of non-attenders, or checklists for structuring the counseling. A final theme was accessibility of services for patients. Examples were the item: “Evening consultation for all patients from vulnerable groups like elderly people, psychiatric patients, people that work long hours, people with low education and single men” (NL) and “walking groups leaving from the general practice” (UK).

### Professional interactions

Presented determinants of practice regarding professional interactions included, for instance, the presence of referral pathways (UK), quality of communication between health professionals (NL), and availability of medical records at interfaces between healthcare providers (GE). Tailored items targeted at professional interactions covered the following themes. A first theme concerned the presence and availability of specific providers in the local setting, such as a fitness trainer (UK) and patient educator (PL). A second theme comprised items to improve communication and teamwork among healthcare providers generally. For instance, specific ideas were “to create meeting points where professionals get to know each other where the services are presented”(NO), “using the network in a national program for improving depression care” (NO), and “enable low threshold for contacts between primary and secondary care” (NO). Connections with municipalities and community organizations, e.g. “weight watchers” (UK; a self help organizations for people who want to lose weight), were also mentioned in this context. A third theme was that a wide range of health professionals could be involved in detection of the targeted chronic condition: “Utilize other caregivers who are involved in care for specific groups as (possibly signaling) entry. Consider homecare, psychiatrist, doctor of nursing home.” (NL). A fourth theme concerned coordination mechanisms, involving individuals or information technology. For instance, items included “Practice nurse as central caregiver, using a concrete protocol” (NL), “Use scannable medication record of the German medical doctors association” (GE). A fifth and final theme was that collaboration had to be included in the role perceptions of healthcare professionals: “Some of GP's tasks are collaboration - but a motivation for collaboration is needed, GPs may use up to 7.5 h per week for this” (NO).

### Incentives and resources

Examples of presented determinants included the availability of devices and staff (PO), financial reimbursement for specific activities (GE), and access to available services (NO). A small number of items was included in this category. A first theme was the item that overall reimbursement of the healthcare provider had to be increased, either as lump sum or as a bonus for good performance. A second theme comprised items to supply specific resources, including staff in the practice, information technology tools, and medical devices. A third theme was that tailored items were proposed for reimbursement (as currently none existed), including telephone consultations (GE), group consultations (NL), longer consultations (NO). A final theme concerned incentives for patients, e.g. for showing up at planned consultations (NL) or vouchers for attending the Weight Watchers (UK).

### Capacity for organizational change

Lack of coordination between municipalities (NO) is an example of a determinant of practice, which was presented to the groups. A few items related specifically to the capacity of organizational change. Most referred to making resources (personal, facilities) available to enable implementation. In addition, there was one item to anchor a new practice in the relevant administrative organization.

### Social, political and legal factors

Only one tailored item was categorized in this domain: publicity for healthcare providers to increase awareness of their existence among potential users (UK).

## Discussion

In the brainstorm interviews, the stakeholders provided many items for interventions and policies to implement evidence-based healthcare for patients with chronic diseases. The items largely mapped onto three domains: individual health professional factors (knowledge, skills, behaviors), patient factors, and professional interactions. Items relating to the knowledge, skills, or behaviors of health professionals comprised by far the largest category, covering both educational strategies and organizational changes. Few items specifically addressed guideline factors, incentives and resources, capacity for organizational change; or wider social, political and legal factors. The relative distribution of items across TICD framework domains was largely similar across different stakeholder groups.

Before elaborating on the findings in several domains of practice, we mention a number of limitations of the study. This international study followed a written study protocol and the fidelity of procedures was monitored during data-collection by the study coordinators. Nevertheless, we could not avoid some differences in the application of the methods, such as different numbers of determinants provided as input for the group interviews or the use of individual interviews in one case. Although we included a range of stakeholders, for practical reasons we did not include patients. This might have reduced the range of items, although the group interviews with patients or their relatives in two countries (NL, NO) did not provide different items than the other groups in those countries. The group interviews were focused on identifying tailored items that could be put into practice, so we might have missed theory-based mechanisms of change. The items are likely to be influenced by the professional disciplines of the participants. For instance, we noticed that no items directly related to healthcare professionals' cognitions, although these are seen as crucial in behavior change psychology. The qualitative analysis required subjective judgments, which we reduced by using a previously developed framework and two independently working researchers. Nevertheless, there is potential bias in the input given at the start of the interviews, the summary of suggestions given by participants and their translation into English. The chosen framework can also be critiqued. For instance, the category “individual professional factors” may be perceived as broad as it covers both educational and organizational interventions. Finally, the relevance of items may be limited to high income countries with a relatively strong primary care system.

The relatively low number of items regarding the clinical guidelines reflects the low number of determinants related to guidelines, which were derived from the previous phase in the TICD project. This may suggest that these were perceived as a given set of valid recommendations. The items regarding the clinical guidelines for chronic conditions called both for clarity and specificity of the guidance (consistent with the view that change requires top-down steering) as well as for the possibility of adaptation to local settings (consistent with the view that change is socially constructed). “Guideline implementability” (the probability that a guideline can be implemented) has received increased attention in recent years [16]. Some aspects of implementability are under the control of guideline developers (e.g. considering co-morbidities, definition of performance indicators), but other aspects have to be largely managed by other decision makers (e.g. local adaptation of national guidelines, organizing resources).

Consistent with frameworks for learning in the work place from the educational sciences [17], the stakeholders had many items to strengthen social interaction during the learning process of healthcare professionals as well as for support and incentives to translate the knowledge learned into practice. This is consistent with current developments in medical education, which emphasize that teaching healthcare providers requires a broad set of competencies [18]. It may be noted that few items of the stakeholders concerned individual cognitions of health professionals, although a large body of research has emphasized the importance of cognitions for behavior change [19]. This may be due to the professional disciplines of the group participants (who were not experts on behavior change), the types of factors we asked them to focus on (not individual cognitions), or such factors being considered but not mentioned as they were considered less relevant for improving chronic illness care.

The large number of items targeted at health professionals' behaviors mainly comprised educational interventions and organizational changes in healthcare, which we interpreted as directly targeted at individual health professionals. Many of the suggested organizational changes directly addressing individual health professionals need to be applied by themselves. Examples include the use of information technology and revision of professional roles. The available evidence supports the idea that such organizational changes can improve quality, efficiency and outcomes of healthcare delivery [20]. It may be noted that we used the domain “organizational capacity for change” for upstream factors only, such as “organizational readiness of change” [21], which can influence individual health professionals indirectly. The low number of such upstream organizational items may reflect the background of the participants. For instance, the inclusion of more senior managers in the groups might have led to more organizational ideas.

A wide range of items focused on involving patients more actively in the healthcare for their chronic condition. Healthcare providers tended to provide the highest numbers of items in this category, which may suggest that they have high expectations of involving patients more actively in chronic illness care. While involving patients actively in their care can serve different purposes, the stakeholders were instructed to focus on items to address a given set of determinants related to a given set of evidence-based recommendations. There is a large literature on patient empowerment, patients' self-management, shared decision making, and related concepts. However, the research evidence that active involvement contributes to better healthcare delivery is limited [22], particularly regarding the use in routine care settings.

While many items were very specific, this was less clear for items regarding professional interactions. While these expressed the idea that teamwork and collaboration of healthcare providers is important for high-quality chronic illness care, the number of tailored items was low. This is consistent with scientific knowledge on the topic. A systematic review found that strengthening of patient care teams can improve quality and outcomes of healthcare, but it was less obvious which factors contributed to team effectiveness [23]. A promising new perspective is offered by social networks analysis, which suggest that the presence of “collaboration behavior” may be related to the structure of healthcare providers' networks [24].

The number of items for financial incentives and resources was relatively low. This was remarkable, because in recent years many programs for improving healthcare have focused on changes in reimbursement of healthcare providers (e.g. pay for performance schemes). In some participating countries (e.g. Netherlands, United Kingdom), reimbursement of healthcare for the targeted chronic conditions is relatively good, so that reimbursement may be no longer the primary concern of stakeholders. It may be noted that the stakeholders had few items regarding incentives or structures in the healthcare system, which may reflect the input that we provided to the group and the position of the individuals involved in the group interviews.

Our study is one of the first comparative studies of methods for tailoring strategies to determinants of practice. Brainstorming in groups of stakeholders proved to be a feasible method to identify many ideas on improving healthcare. It is useful to know that different stakeholders provided similar types of items (in terms of TICD framework domains addressed). If resources are limited, it may be advisable to include at least health professionals, because they appeared to be highly productive in the interviews. Another implication of this study is that prioritization of items is required, given the high number of items, when designing an implementation program.

As our study is one of the first of its kind, it is important that more comparative studies are done to develop and test methods for tailoring strategies to determinants for improving healthcare. We used group interviews to match strategies to determinants of practice, but a range of other methods is available that can potentially be used for this purpose. These include pragmatic survey and interview methods as well as methods that are more strongly guided by theories on change, such as intervention modeling [25]. The effectiveness of a tailored implementation strategy resulting from a tailoring method is the ultimate outcome of interest, but future evaluations are likely to rely on intermediate outcomes like we did. The validity of such intermediate outcomes needs attention, because it is difficult to assess the plausibility of items in tailoring exercises.

## Supporting Information

Data S1 (XLSX)
